# The RNA editing patterns are different in blood of euthymic and depressed bipolar patients

**DOI:** 10.1192/j.eurpsy.2023.1200

**Published:** 2023-07-19

**Authors:** M. Hayashi, N. Salvetat, C. Cayzac, F. J. Checa-Robles, C. Lozano, J.-D. Abraham, B. Dubuc, S. Mereuze, J. V. Nani, F. Molina, E. Brietske, D. Weissmann

**Affiliations:** 1 UNIFESP, SAO PAULO, Brazil; 2 ALCEDIAG/Sys2Diag, Montpellier, France; 3 Queen’s University School of Medicine, Kingston, Canada

## Abstract

**Introduction:**

Bipolar disorder (BD) is a severe mental disorder associated with functional impairment, high disability and premature mortality. Modifications of editing in mRNA of serotonin receptor subtype 2C (5-HTR2c) was reported by us in depressed suicide decedents. We have also identified a panel of RNA editing-based blood biomarkers for the diagnosis of BD, which also allowed to discriminate unipolar depression from BD with high sensivity and specificity.

**Objectives:**

Herein, aiming to confirm the diagnostic value of this panel, a new cohort of BD patients was recruited in Brazil.

**Methods:**

This study is based on the analysis of 47 control patients (CTRL) compared to 40 patients with bipolar disorder (BD). BD patients (BP) were classified into 4 subgroups: euthymic (BP_EUT, n = 17), depressive (BP_DEP, n = 11), manic/hypomanic (BP_HM, n = 7) and mixed (BP_MIX, n = 5). The diagnostic value of a panel of RNA editing-based blood biomarkers for the diagnosis of BD, which includes a set of eight genes, namely PDE8A, CAMK1D (calcium/calmodulin-dependent protein kinase type 1D); GAB2 (growth factor receptor bound protein 2-associated protein 2); IFNAR1 (interferon alpha/beta receptor 1); KCNJ15 (ATP-sensitive inward rectifier potassium channel 15); LYN (tyrosine-protein kinase Lyn); MDM2 (E3 ubiquitin-protein ligase Mdm2); PRKCB (protein kinase C beta type), which was able to discriminate unipolar depression from BD with high sensivity and specificity, was confirmed here by testing an independent cohort of patients suffering from BD recruited in a well-known genetic admixed ancestry population, which is typical in South America, more specifically in Brazil.

**Results:**

We identified new combinations allowing a clear discrimination of euthymic versus depressed bipolar patients, and euthymic versus healthy controls, confirming that RNA editing is a key mechanism in the physiopathology of mental disorders, in particular in BD.

**Conclusions:**

In conclusion of this study, we confirm that RNA editing is a key mechanism in the physiopathology of mental disorders in general, and in BD in particular, and that measuring changes in this mechanism at the peripheral level allowed us to stratify BD patients not only with respect to their symptomatology, but also with respect to the pathophysiology, thus paving the way for personalised medicine in psychiatry.

**Disclosure of Interest:**

None Declared

